# The Effectiveness of Treating Osgood–Schlatter Disease (OSD) with Leukocyte-Rich Platelet-Rich Plasma (LR-PRP) Depending on the Duration of the Disease

**DOI:** 10.3390/jcm13144220

**Published:** 2024-07-19

**Authors:** Tomasz Guszczyn, Monika Kulesza, Grzegorz Maciąg, Aleksandra Kicman, Sławomir Ławicki

**Affiliations:** 1 Department of Pediatric Orthopaedics and Traumatology, Medical University of Bialystok, 15-274 Bialystok, Poland; 2 Department of Population Medicine and Lifestyle Diseases Prevention, Medical University of Bialystok, 15-269 Bialystok, Poland; monika.kulesza@sd.umb.edu.pl (M.K.);; 3 Department of Aesthetic Medicine, Medical University of Bialystok, 15-267 Bialystok, Poland; olakicman@gmail.com

**Keywords:** Osgood–Schlatter disease, leukocyte-rich platelet-rich plasma, VAS scale, Tegner scale, Lysholm scale, KOOS scale

## Abstract

**Background**: Osgood–Schlatter disease (OSD) occurs mainly in physically active adolescents, causing significant physical activity restrictions. The aim of this study is to compare the effectiveness of treating OSD with leukocyte-rich platelet-rich plasma (LR-PRP) depending on the duration of the disease and to attempt to develop an alternative treatment method to the currently used conservative therapy. **Methods**: Treatment efficacy was evaluated using the VAS, Tegner, Lysholm, and KOOS scales. Subject satisfaction, return to sports activity, potential adverse effects, and X-ray evaluation were likewise used to assess the success of the procedure. **Results**: Analysis across all scales showed statistically significant treatment effectiveness with LR-PRP in both groups of patients. When comparing the two groups, significantly better treatment outcomes were achieved in the acute phase of OSD. Treatment satisfaction in the acute OSD group was 95%, compared to 64% in the chronic group. The MCID value after LR-PRP injection in acute OSD compared to chronic OSD reached 100% vs. 81% on the VAS scale, 95.5% vs. 55% on the Tegner scale, 95% vs. 47% on the Lysholm scale and 91% vs. 27% on the KOOS scale. No adverse effects were recorded in either group. **Conclusions**: The high efficacy of LR-PRP treatment in patients with acute OSD, in correlation with high safety, as well as rapid and lasting results, can be an effective and beneficial alternative to conservative treatment. This single procedure seems particularly justified in a group of young professional athletes, where absence from training can lead to serious consequences.

## 1. Introduction

Physical activity is essential for the proper development of the musculoskeletal system in children. Participation in sports can help children maintain strong bones and muscles and improve cardiovascular health. Sports enhance social skills: they provide children with opportunities to interact with their peers and make new friends, and they also teach teamwork. Regular participation in sports boosts self-esteem and improves mental health.

Despite the many benefits of sports for children, they can also cause negative consequences. According to the Centers for Disease Control and Prevention (CDC), over 2.6 million children under the age of 19 in the US are being treated in emergency departments every year for sports- and recreation-related injuries. In addition to typical sports-related injuries, physical activity can lead to overuse injuries, which occur more frequently than acute injuries and are trending upward, especially among fast-growing adolescents [1,2]. In this population, one of the most common overuse injuries is apophysitis. One of the most common conditions in this group is apophysis of the tibial tuberosity, known as Osgood–Schlatter disease (OSD) [3]. About 10% of adolescents are affected by OSD [4].

Osgood–Schlatter disease mainly affects highly physically active children between the ages of 8 and 15, especially those doing intensive sports training [5,6]. The etiology of OSD is multifactorial, and there are still many theories attempting to explain the pathogenesis of this disease. Due to chronic overload, numerous micro-injuries occur in the tibial tuberosity area, contributing to inflammation and pain in the tibial tuberosity area [5,7].

The main symptom of OSD is pain over the tibial tuberosity, which eliminates or significantly limits the child’s sports activity. The pain is caused by inflammation in the area where the patellar ligament attaches to the bone. According to studies on rats and mice, overexertion activities with eccentric contractions cause structural disruption of the enthesis, which is where the fibrocartilaginous layer begins to dominate [8,9]. The consequence of a prolonged process in this region is the formation of a loose bone fragment, which is evidence of chronic OSD. The prevailing view is that OSD is self-limiting and lasts from 6 to 18 months [10,11]. According to other sources, the symptoms can last up to 2 years, which may directly cause young athletes to completely stop participating in sports [12]. In a systematic review conducted by Neuhaus C et al., after analyzing 731 original and updated search records, as well as an additional 37 records from other sources, it is stated that OSD is a long-lasting pain condition that occurs during puberty and can develop into chronic knee pain [13].

The treatment of OSD depends on the duration of the disease and the bone maturity of the patient, particularly with the presence of an open growth plate in the proximal tibia. If the growth plate is closed, surgical methods to remove the tuberosity, either open or arthroscopic, can be used. Both methods are highly effective, but due to their invasiveness, they are not without side effects [14,15]. When the growth plate is open, only conservative treatment methods are available. Traditional conservative treatment involves stopping or modifying physical activity, rehabilitation, or temporary immobilization. Rehabilitation treatment is highly effective, reaching over 90%, but, unfortunately, it is time-consuming. According to some protocols, it can last 6–12 months [11,16]. An alternative to this type of therapy may be injecting the tuberosity. Currently, cortico-steroid injections are not recommended due to the increased risk of atrophy and subsequent rupture of the patellar tendon [17]. An effective and safe alternative to steroidal anti-inflammatory drugs is a hyperosmolar dextrose solution. Dextrose injections are a form of prolotherapy, where the increased concentration of glucose in the extracellular matrix stimulates the synthesis of growth factors such as TGF-beta, VEGF, IGF, and FGF [18,19]. The effectiveness of dextrose in the treatment of OSD was first described by Topol in 2011 [20]. Subsequent reports confirm the effectiveness of dextrose in the therapy of OSD [21].

It seems that an even more effective method of accelerating healing may be the administration of autologous growth factors. A simple and inexpensive method using the action of peptide growth factors is the preparation of PRP (platelet-rich plasma). It exhibits a strong anabolic effect, thereby accelerating healing and regeneration processes [22]. In addition, numerous cytokines contained in leukocyte-rich platelet-rich plasma (LR-PRP) have been shown to strongly stimulate collagen biosynthesis [23] and promote the proliferation of various cells [24,25]. In vitro studies have also demonstrated potent anti-inflammatory effects of LR-PRP through COX-2 inhibition [26]. These effects explain the increasing use of PRP in many fields of medicine, from aesthetic medicine to maxillofacial surgery, dentistry, general surgery and orthopedics. In the latter, PRP is used for a variety of conditions, including osteoarthritis, tendonitis, ligament injuries, muscle strains and chronic degenerative conditions [27]. The efficacy of LR-PRP injections has been confirmed for lateral epicondylitis and patellar tendinopathy, while leukocyte-poor platelet-rich plasma (LP-PRP) has been confirmed for osteoarthritis of the knee and PRP injections for plantar fasciitis and pain elimination at the site of BTB ACL patellar tendon graft donation [28].

Two cases of successful use of PRP in patients with OSD have been reported in the literature [29,30]. In 2023, we published the results of our study that showed high efficacy of LR-PRP in patients with chronic OSD [31]. In addition, the results of statistical analysis showed significantly higher treatment efficacy with LR-PRP at an earlier stage of the disease. As is well known, OSD primarily affects young, ambitious athletes who are forced to interrupt their training due to the condition. A long period of absence, especially at the beginning of a career, can lead to complete elimination from sports and cause emotional problems during development. For this reason, finding a fast, effective and safe method of treating OSD during the period of intense growth in young athletes seems very necessary. Therefore, we decided to compare the results of treatment using LR-PRP in patients with acute and chronic OSD. Our hypothesis is that the use of LR-PRP in the acute form of OSD will be more effective than in the chronic form, and, due to its rapid action, it may be an alternative to conventional treatments for OSD.

## 2. Materials and Methods

### 2.1. Patient Characteristics

The characteristics of the study group are shown in Table 1. The current study included 150 patients who came with symptoms of pain in the knee area to the Department of Orthopedics and Traumatology of the Children’s Clinical Hospital in Bialystok. These patients were diagnosed with Osgood–Schlatter disease based on a history of reduced sports activity due to pain in the tibial tuberosity area. The inclusion criteria for these patients were the clinical symptoms of Osgood–Schlatter disease and/or a characteristic image of the disease in an X-ray examination. Clinical examination of the patients revealed pain, swelling and/or hypertrophy in the tibial tuberosity area. X-ray images showed enlargement of the tibial tuberosity outline and/or loose bone fragments at the patellar ligament attachment site. The patients were divided into the following two groups based on the duration of symptoms: acute form (duration of symptoms up to 12 months) and chronic form of the disease (duration of symptoms more than 24 months). Criteria for exclusion included previous knee injuries, other conditions of the knee joint and concurrent medical conditions (especially rheumatologic and endocrinologic conditions) and use of nonsteroidal anti-inflammatory medications in the past 14 days.

Patients were treated with conservative methods, such as rehabilitation, immobilization of the limb and NSAID drugs (nonsteroidal anti-inflammatory), prior to PRP injection; however, these methods did not result in the desired treatment effects. Table 2 includes information on the percentage of patients with chronic and acute OSD under each treatment modality.

### 2.2. LR-PRP Preparation

The LR-PRP preparation is obtained by drawing 40 mL of venous blood from the patient’s elbow vein. This blood is collected into a syringe that contains 1 mL of an anticoagulant, specifically sodium citrate (S-Monovette; 10 mL 9NC:0.106 mol/L). The blood is then centrifuged at 3000 rpm for 10 min at room temperature. This process results in a supernatant with two layers: the upper layer is platelet-poor plasma and the lower layer is leukocyte-rich platelet-rich plasma (LR-PRP). The final yield of the LR-PRP preparation is approximately 2.5–3 mL. The median number of platelets per cubic millimeter was found to be 982.4 ± 298 for LR-PRP and 208.4 ± 78 for venous blood (VB). The median concentration of blood cells per cubic millimeter was 9.9 ± 4.9 for LR-PRP and 5.4 ± 2.4 for VB. All these procedures were carried out under sterile conditions.

### 2.3. Treatment Procedure

Therapeutic management in the study group included injecting the tibial tuberosity with platelet-rich preparation. LR-PRP was injected at a volume of 1–1.5 mL into the tibial tuberosity area in the supine position. The needle was positioned into the area of the tibial tuberosity, which the patient identified as the most tender point. To avoid diluting the LR-PRP preparation, the injection site, which had been prepared beforehand, was anesthetized on the surface with ethyl chloride. A 23G × 1 ¼” needle was used to puncture the area of the tuberosity at the site of the distal attachment of the patellar ligament to the bone. The needle was then slightly withdrawn (1–2 mm) to deposit the LR-PRP in the fibrocartilaginous tissue of the patellar ligament attachment approximately 1–2 cm proximal to the growth cartilage. Patients did not use NSAID drugs before and during treatment. The patient’s knee was immobilized in an orthosis with approximately 15-degree flexion for a period of 2 weeks, with a restriction of sports activities for a total of 6 weeks after the injection. After the removal of the orthosis, patients were able to walk without restriction and then gradually increase their exertion. After a period of 6 weeks, the patient was able to return to full sports activity. If there was a need for another LR-PRP injection, the patient’s knee was given a re-injection no sooner than 3 months following the initial procedure.

### 2.4. Outcome Evaluation

The assessment of knee function prior to the LR-PRP injection and following the follow-up period was conducted using the Lysholm Knee Scoring Scale (LKSS), Tegner Activity Scale, and Osteoarthritis Outcome Score (KOOS). The evaluation of pain was carried out using the VAS scale [32,33]. The VAS scale was assessed as pain during or immediately after physical activity.

Information from all the utilized tests (Lysholm, Tegner, KOOS, and VAS) was gathered through the completion of their paper versions under the supervision of a physician. In both study groups, the follow-up period was an average of 36 months (min 24 months, max 72 months, S.D. 10.72). Patients completed the forms based on their personal feelings. In addition, X-ray images were taken both prior to the PRP injection and following the observation period.

### 2.5. Statistical Analysis

A statistical analysis of results was performed. Due to significant deviation from normality of distribution of all continuous variables examined with the Shapiro–Wilk test, a Mann–Whitney U-test was performed to assess the differences between the studied groups and a Wilcoxon signed-rank test was used to assess differences between pre- and post-injection values in each group. For categorical variables, a Fisher’s exact test was used. For analysis of the interaction between the groups and the satisfaction rate on the MCID-achievement rate, a Cochran–Mantel–Haenszel test (CMH) with a Zelen’s exact test was performed. A Fisher’s exact test with a Bonferroni correction was used as a post hoc test. The significance level was set at an alpha value below 0.05. All statistical analyses were conducted using SAS software, Version 9.4, for Windows (SAS Institute Inc., Cary, NC, USA).

### 2.6. Ethical Approval

This study was approved by the local ethics committee of the Medical University of Bialystok (R-I-002/33/2015). Written consent was obtained from the parents/legal guardians of the minor patients before the study.

## 3. Results

### 3.1. Satisfaction with Treatment and Return to Activity

**Figure 1:**
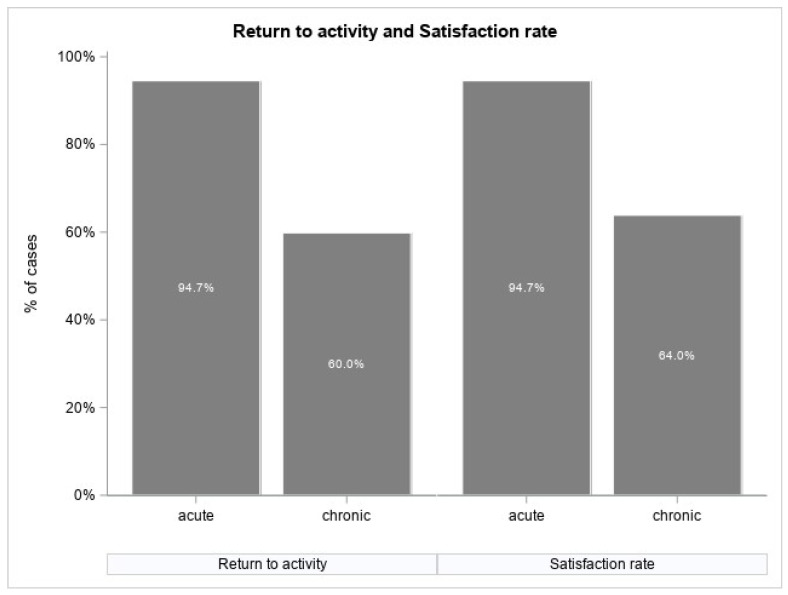
Bar chart presenting percentage of patients that declared return to full physical activity (**left**) and self-reported satisfaction (**right**) for each of the studied groups.

Our study showed that 94.7% of subjects with acute forms of OSD were satisfied with the results obtained from the treatment procedure, while 64% of subjects in the group with chronic forms of the disease were satisfied with the treatment. Moreover, 94.7% of patients with the acute form of the disease returned to full physical activity. Four patients (5.3%) stopped participating in competitive sports at their parents’ urging but successfully participated in school sports activities without restrictions. In the group of patients with chronic OSD, 60% of respondents returned to full physical activity (Figure 1). Among the patients from the chronic OSD group who were not satisfied, 10 (which is 13.3% of the chronic OSD group) chose to undergo surgical treatment. They had surgery after the growth cartilage fusion of the tibial tuberosity was achieved through an arthroscopic bursectomy. These patients had repeat injection procedures at 3 and/or 6 months without achieving satisfactory results.

### 3.2. Evaluation of Knee Pain and Function in VAS, Tegner, Lysholm and KOOS Scales

There was a significant change in value of every scale analyzed in the study within both of the groups. 

Analysis of the VAS scale showed a statistically significant reduction in the median scores obtained in patients with the acute and chronic forms of OSD after PRP injection of the tibial tuberosity (acute: 0 {0–1}, *p* < 0.0001; chronic: 1 {0–3}, *p* < 0.0001). The difference in the median value of VAS scale before and after the injection was statistically significant between patients with acute and chronic forms of OSD (6 {5–7} and 5 {3–6}, *p* < 0.0001, respectively). 

Analyzing the score of the median values of the Tegner scale, we observed statistically significant improvement in both of the groups after PRP injection (acute: 7 {6–8}, *p* < 0.0001; chronic: 6 {5–8}, *p* < 0.0001). There was also a significant difference between the groups in regard to median change of Tegner scale (acute: 1 {1–1}, *p* < 0.0001; chronic: 1 {0–1}, *p* < 0.0001).

The group of patients with an acute form of OSD had significantly lower pre-injection and higher post-injection values in the Lysholm and KOOS scales than the group with the chronic form (Lysholm: 77 {70–82} vs. 88 {82–90}, *p* < 0.0001 and 95 {95–100} vs. 95 {91–95}, *p* < 0.0001; KOOS: 78 {72–80} vs. 88 {80–90}, *p* < 0.0001 and 99 {97–100} vs. 95 {90–98}, *p* < 0.0001, respectively). The results are shown in Table 3.

### 3.3. The Minimally Clinically Important Difference (MCID)

**Figure 2:**
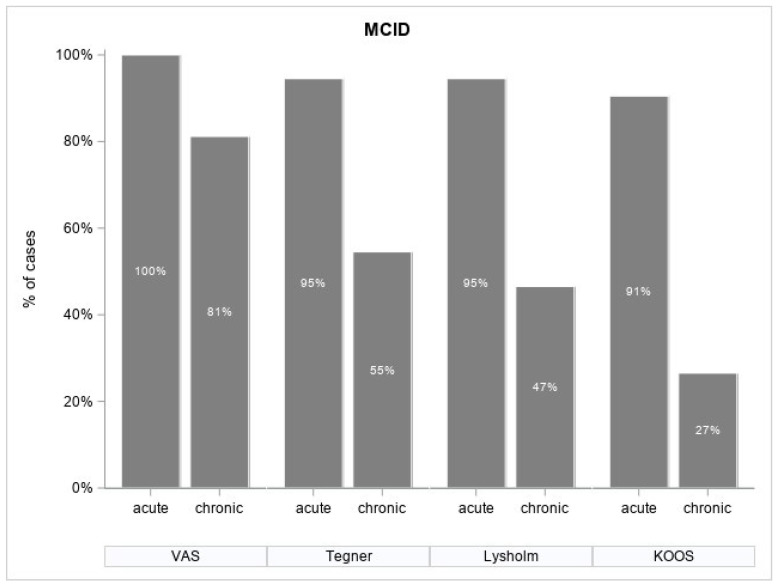
Bar chart presenting percentage of patients’ MCID-achievement rate for VAS, Tegner, Lysholm and KOOS scales in each of the studied groups.

The Minimally Clinically Important Difference (MCID) for the VAS, as suggested by data from populations of adult rheumatology patients, is considered to be a decrease of 1.37 [34]. In our research, the Minimal Clinically Important Difference (MCID) for the VAS scale, which is at least two points, was reached by all patients (100%) with acute OSD and in 81% with chronic OSD. The MCID values for the Tegner, Lysholm and KOOS scales, as evaluated by Qiao in the context of knee pathology, are as follows: for the Tegner scale, MCID is 0.9; for the Lysholm scale, MCID is 11.1; and for the KOOS scale, MCID is 10.0 [35]. In reference to the data above, our study found that an MCID greater than 0.9 points on the Tegner scale was achieved by 95.5% of patients with acute OSD and 55% of patients with chronic OSD. An MCID greater than 11.1 points on the Lysholm scale was reached by 95% of patients with acute OSD and 47% of those with chronic OSD. Lastly, an MCID greater than 10 points on the KOOS scale was attained by 91% of patients with acute OSD and 27% of patients with chronic OSD. The percentage distribution of patients’ outcomes exceeding MCID values in the studied scales is shown in Figure 2.

There was a significant association between the rate of satisfaction among patients and the rate of MCID achievement across all tested variables controlled for the duration of the symptoms (*p* < 0.0001 in all cases). The aforementioned association variances were heterogeneous between the studied groups for change in both the Lysholm and KOOS scales (*p* = 0.0163 and *p* = 0.0159, respectively). A post hoc analysis with a Fisher’s exact test was performed with the alpha level set at 0.0083 after Bonferroni correction. In almost each stratum, there was a statistically significant correlation between satisfaction and the MCID achievement rate of the investigated groups (shown in Table 4). One of the exceptions was the change in VAS scale for patients with acute OSD due to lack of patients who did not achieve MCID values. The second one were patients with chronic OSD in regard to change in the Lysholm scale, which showed a possible trend for significant association between given parameters (*p* = 0.0086). The results are shown in Table 4.

### 3.4. Radiological Evaluation

**Figure 3:**
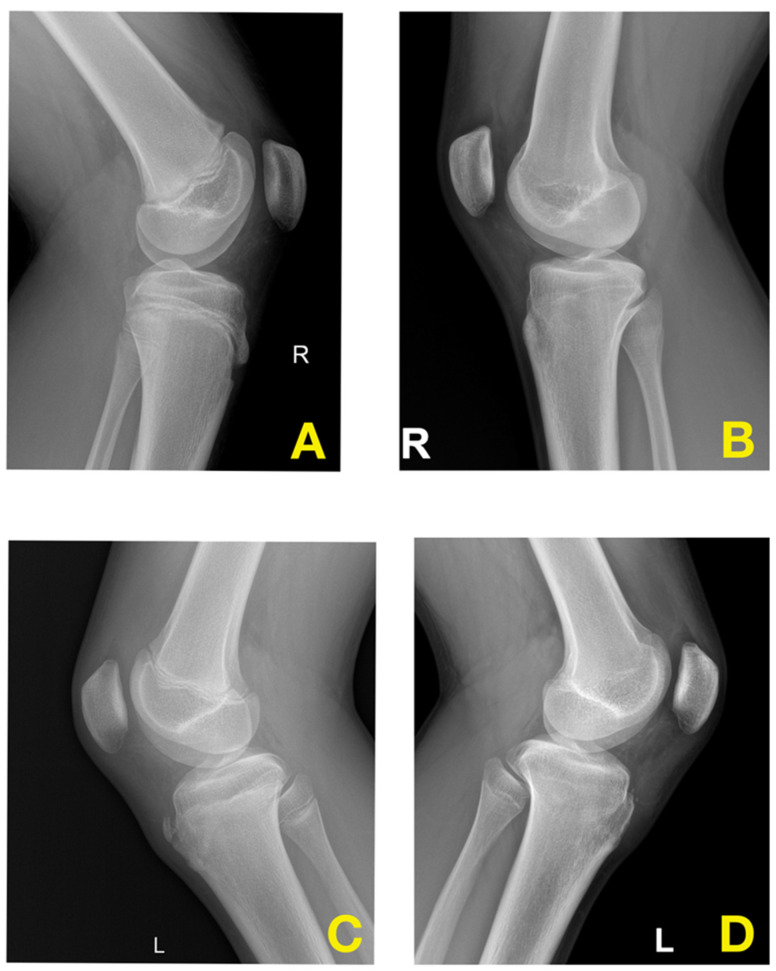
The morphology of the tibial tuberosity area: X-ray images of a patient with acute OSD before (**A**) and after LR-PRP injection (**B**) and of a patient with chronic OSD before (**C**) and after injection (**D**) after an observation period of 3 years.

In both patient groups studied, we did not observe any complications. Radiological examinations performed before and after treatment showed no changes. In patients with the acute form of OSD who did not initially have a loose bone fragment, there was no formation of such a fragment after the completion of treatment (Figure 3A,B). In other cases, no changes were observed in the morphology of the tibial tuberosity area (Figure 3C,D).

## 4. Discussion

The most important finding of the presented study is that 95% of patients with acute form of OSD achieved a full return to sport activities in comparison to only 60% of the patients with a chronic form of OSD reporting satisfaction and 64% achieving a return to sport activities after one PRP injection and 6 weeks follow-up. 

OSD is considered a self-limiting disease after a 12–18-month observation period with physical activity restriction, with a good clinical response to conservative treatment in 80–90% of patients, depending on the study [36]. On the other hand, Krause et al. [37] demonstrated that, even without any treatment, 76% of patients with OSD experienced no activity limitations. However, it is worth noting that 60% of these patients still reported discomfort when kneeling [37]. Due to children performing intensive sport activities being most commonly affected, conservative treatment ongoing for 12–18 months is frequently not acceptable for patients, as it prevents them from performing physical activity with their prior intensity level. 

It is particularly important to create both an efficient and effective therapeutic approach in this group of children. Currently, unloading and rehabilitation are recommended [6]. Gerulis et al. [16] showed that combining physical activity restriction with physiotherapy resulted in the disappearance of symptoms after 13 months of treatment. This is in contrast to the 15 months it took for symptoms to disappear when only physical activity restriction was implemented [16]. Strickland et al. [38] conducted a pilot study with 25 patients with OSD suffering from symptoms for 8 months on average. Physiotherapy treatment consisted of a myofascial release massage and stretching of the quadriceps group, with 90% of the patients returning to physical activity after a mean time of treatment of 20 days [38]. Bezuglov et al. [11], in a study on 28 patients with OSD, reported that kinesiotherapy and physiotherapy being performed for 6 months was an effective treatment in 96% of the patients, with approximately 36% of patients reporting discomfort upon reassuming of sport activity, which led to restriction of the activity [11]. The question of the effectiveness of rehabilitation as a treatment for OSD could be evaluated in a study that would compare the effectiveness of a novel self-management approach versus the usual care in enhancing self-reported knee-related functions in sports [39].

The results of our study suggest that the effectiveness of LR-PRP treatment is similar or higher than that achieved with rehabilitation methods. Furthermore, it has a significant advantage in that it is a one-time procedure. Fast intervention is a key factor for young athletes. In rare cases of failure of the first injection, PRP can be reapplied. In the group with acute OSD, no patient decided to have the injection repeated. Four patients who were dissatisfied with the treatment decided to cease participation in competitive sports after their parents’ suggestions. However, they still participated in PE classes without restrictions. In this aspect, it should be noted that, in the acute OSD group, 100% of patients achieved a reduction in pain on the VAS scale of >2 degrees, defined as MCID.

An alternative to LR-PRP in the treatment of OSD may be the use of dextrose, already described by Topol et al. in 2011 [20]. In this research, 54 athletes and 65 knees were categorized into three groups: those receiving usual care treatment, those receiving lidocaine/dextrose injections, and those receiving lidocaine-only injections. The findings indicated that both injection groups (dextrose/lidocaine and lidocaine-only) had a higher incidence of unaltered sport. Furthermore, the capacity to participate in sports activities without symptoms was greater in patients who received dextrose/lidocaine injections compared to those who received usual care (which included physical therapy) or lidocaine-only injections. In this study, patients were qualified for the study after 3 months of unsuccessful rehabilitation, and the scheme included 3 injections with monthly intervals. Pain reduction and return to sport were reported after 3 and 12 months. Our study shows similar results; however, there were two major differences in regard to patients’ characteristics. Firstly, patients in our study had longer durations of the disease, equal to 6 months in the acute group and 36 months in the chronic group vs. 3 months in the Topol et al. [20] study. Secondly, follow-up in our study continued for 3 years in comparison to 1 year in the study by Topol et al. [20]. In this time period, the median change in Tegner scale was 1 point, which shows objective improvement in the physical activity of the patients. 

The work by Nakase et al. [40], which negates the effectiveness of dextrose in OSD due to incorrectly chosen statistical methods, should not be used as an argument in the discussion. Further support for the effectiveness of dextrose treatment is also provided by a study published by Wu et al. [21]. This 2022 double-blind randomized controlled trial, which studied 22-year-old patients, showed markedly improved results following the administration of dextrose compared to the administration of saline. The studied outcome was a VISA-Patella Score, with statistically significant and clinically important changes in both of the studied groups after a follow-up of 3 months (with a mean difference of 25.4 (22.4 to 28.3); *p* < 0.0001), 6 months (mean difference 6.2 [3.2 to 9.4]; *p* < 0.0001) and 12 months (mean difference 5.5 [1.9 to 9.1]; *p* = 0.0026).

Hyperosmolar dextrose injection, also described as a possible treatment for OSD, is a kind of injection-based treatment used to relieve musculoskeletal pain; it is known as prolotherapy. It is regarded as regenerative injection therapy, triggering an inflammatory response at the injection site, which induces fibroblast proliferation and subsequent collagen formation [41]. Hyperosmolar dextrose solution has been shown to stimulate the synthesis of peptide growth factors, which may be responsible for clinical effects [18,19]. Platelet-rich plasma (PRP) therapy appears to be the next generation of prolotherapy. In addition to the aforementioned effect of peptide growth factors, PRP contains numerous cytokines that modulate the inflammatory process [42]. In vitro studies have shown strong stimulation of collagen biosynthesis both under standard cell culture conditions and under induced inflammatory states [23,26]. These biological effects may explain the strong and lasting therapeutic effect in the course of acute OSD, where inflammation is the dominant component.

The main symptom of OSD is localized pain in the projection of the tibial tuberosity; therefore, the VAS scale may be one of the accurate indicators of treatment effectiveness. Analysis of the average pain reduction values on the VAS scale by 6 pts in the acute form group and 5 pts in the chronic form group indicates a high effectiveness of LR-PRP injections. However, only the MCID achieved on the VAS scale shows us results more closely correlated with patient satisfaction. In the acute OSD group, MCID was achieved in 100% of patients, while, in the chronic group, it was achieved in 84%. These results seem to correlate more closely with treatment satisfaction (94% in acute OSD and 64% in chronic OSD) than the VAS scale reduction alone. 

In this study, scores of functional scales (Lysholm, KOOS) and the Tegner Activity Scale for Sports were collected to measure the ability to return to sports. Both Lysholm and KOOS scales reflect the general condition of the joint, with pain being only a minor component of the scores. However, these results can objectively show the recovery of knee function and the possibility of a full return to sports.

An increase by 18 points on the Lysholm scale in patients with acute OSD seems to be associated with a high percentage of MCID achievement (95%) and is very similar to the return to sports activity rate (94%). Similarly, an increase by 20 points on the KOOS scale was associated with MCID achievement in 91% of the patients. These scores are significantly lower in patients with the chronic form, with an increase on the Lysholm scale by only 5 points being associated with an MCID achievement rate of only 47% in patients and an increase of 9 points on the KOOS scale and an MCID being achieved by 27% of patients.

The Tegner Activity Scale for Sports was another scale analyzed in our study. This scale shows the strongest correlation with satisfaction and the ability to return to sports. A post-injection median value of 7 in th acute OSD group and a median value of 6 for the chronic OSD group was obtained. These results were associated with an MCID achievement rate of 95% and 55% in those groups, respectively.

The results of the functional scales (Lysholm and KOOS), and especially the Tegner Activity Scale for Sports, allow us to objectify patients’ ability to return to sports. Pain, being the main symptom of OSD, can be subject to a large margin of error when evaluated by the VAS scale alone. Therefore, we believe that only correlated results of the VAS scale, functional scales, and the sports activity level can objectively assess the effectiveness of OSD therapy.

The correlation between achieving MCID on the functional scales and the VAS scale with treatment satisfaction shows both statistically and clinically significant improvement in both groups. However, the degree of association between treatment satisfaction and objective functional scales appears to be more dependent on patients with a short duration of illness. This suggests that in the early stage of the disease, it is possible to achieve positive clinical outcomes confirmed by good results in the applied functional tests. This indicates the validity of earlier qualification for OSD treatment. The results of our study suggest that claims about OSD being a self-limiting disease in 80–90% of patients lead to unnecessary delays in therapy. 

Our results also show a positive effect of PRP injections in patients with a chronic form of the disease, but it seems to not be as easily perceptible in regard to association in terms of both improvement in functional outcomes and overall patient satisfaction as in acute OSD.

Interesting insights into the treatment of OSD are provided by the work of Smida M et al. from 2018, which found a reduced level of vitamin D3 in patients with OSD and high treatment efficacy with supplementation of this vitamin [43]. Undoubtedly, the administered dose of 6000 IU per day of vitamin D for 6 or 12 months can modify the course of OSD. The results of this treatment method indicate an efficacy of 84% (80 knee joints out of 95 treated) after 3 months of treatment and 95% (90 knee joints out of 95 treated) after one year [43]. It is difficult to compare these results with ours, partly because we do not have information on whether the treatment with vitamin D was applied in the acute or chronic phase of OSD. We see the advantage of LR-PRP therapy in the shorter treatment duration (6 weeks vs. 3–12 months) and the local administration of the preparation in a disease that occurs in 90% of cases as a single-joint condition.

An important aspect of LR-PRP injections in our study was the lack of the side effects. Although it is an invasive procedure involving breaking integument continuity, it seems that the autologous nature of the preparation and the high leukocyte content reduce potential complications in regard to ossification or infection. The simple preparation method and the low cost of treatment are other advantages of this therapy.

One problem reported by our patients was short-term pain, usually lasting up to 30 min after the injection itself. Patients typically left the ward after 2–3 h; after this period, they did not report any spontaneous pain. Immobilization of the knee in a brace allowed for full weight-bearing without discomfort.

We are aware of the limitations of this work, which include the lack of a comparison group treated with another injection method and the use of MCID values for the studied scales from other knee joint conditions. We are currently in the process of collecting data from a study comparing the effectiveness of LR-PRP with an injection of saline solution with Lignocaine.

## 5. Conclusions

We believe the LR-PRP can be successfully used as a treatment regardless of duration of the symptoms of OSD, as it seems to be an effective and efficient alternative to rehabilitation due to its quick and lasting effect and relatively safe procedure. The possibility of introducing a fast and convenient form of treatment consisting of a single appointment and a single procedure is an important advantage in rehabilitation in regard to duration of treatment (6 weeks vs. 3–12 months). A single LR-PRP injection seems to be particularly justified in the group of young athletes engaged in sports at a competitive level, as a long absence from training can have serious consequences on their sporting career and the child’s emotional sphere.

## Figures and Tables

**Table 1:** General characteristics of the study group. Data are presented as median and interquartile range (Q1–Q3) and frequencies depending on characteristics of the data. Knee is meant as the knee affected by the condition. *-red color indicates statistically significant results.

	OSD Group		
	<12 Months (n = 75)	>24 Months (n = 75)	*p*-Value
Age (year)	IQR: 11–13 Median: 12	IQR: 14–16 Median: 14	-
Duration of symptoms (months)	IQR: 3–9 Median: 6	IQR: 30–48 Median: 36	-
Age at illness onset	IQR: 10.75–12.25 Median: 11.25	IQR: 10.5–12 Median: 11	>0.05 *
Gender	Male—58 Female—17	Male—53 Female—22	>0.05 *
Knee	Right—63 Left—12	Right—56 Left—19	>0.05 *

**Table 2:** Percentage of patients in conservative treatment. *-red color indicates statistically significant results.

	<12 Months (%)	>24 Months (%)	*p*
Immobilization	14.7	17.3	0.656
Rehabilitation	76	58.7	0.024 *
Cessation of physical activity	70.7	61.3	0.228
NSAID drugs	16	58.7	<0.005 *
Lack of treatment	8	14.7	0.198

**Table 3:** Comparison of functional scale scores in patients divided according to duration of symptoms. Data are presented as median (lower and upper quartile).

		Acute OSD Group (Median)	Chronic OSD Group (Median)	*p*-Value (between Groups)
VAS Scale	Pre-injection	7 (6–8)	7 (6–8)	0.87
	Post-injection	0 (0–1)	1 (0–3)	<0.0001
	Diff	6 (5–7)	5 (3–6)	<0.0001
	*p*-value (within groups)	<0.0001	<0.0001	-
TEGNER Scale	Pre-injection	6 (5–7)	5 (5–7)	0.3191
	Post-injection	7 (6–8)	6 (5–8)	0.0028
	Diff	1 (1–1)	1 (0–1)	<0.0001
	*p*-value (within groups)	<0.0001	<0.0001	-
LYSHOLM Scale	Pre-injection	77 (70–82)	88 (82–90)	<0.0001
	Post-injection	95 (95–100)	95 (91–95)	<0.0001
	Diff	18 (26–13)	5 (10–4)	<0.0001
	*p*-value (within groups)	<0.0001	<0.0001	-
KOOS Scale	Pre-injection	78 (72–80)	88 (80–90)	<0.0001
	Post-injection	99 (97–100)	95 (90–98)	<0.0001
	Diff	20 (17–28)	7 (4–14)	<0.0001
	*p*-value (within groups)	<0.0001	<0.0001	-

**Table 4:** Comparison of frequency of functional scale scores in regard to MCID achievement in patients divided according to duration of symptoms. CMH and Zelen’s tests’ *p*-values are shown to the left and Fisher’s exact test *p*-values are reported to the right of the Satisfaction-MCID tables. Relative risk for each of the strata was reported as effect-size estimator.

Parameter	Duration of Symptoms (Months)	*p*-Value of CMH Test (Zelen’s Test)	Patient Reported Satisfaction	N Achieved MCID (%)	N MCID Not Achieved	RR	Fisher’s Exact *p*-Value (α = 0.083)
VAS Scale	<12	<0.0001 (-)	No	4 (5.3%)	0	—	(-)
			Yes	71 (94.7%)	0		
	>24		No	13 (17.3%)	14 (18.7%)	2.08 (1.47–3.87)	<0.0001
			Yes	48 (64%)	0		
TEGNER Scale	<12	<0.0001 (0.1285)	No	0	4 (5.3%)	—--	<0.0001
			Yes	71 (94.7%)	0		
	>24		No	2 (2.7%)	25 (33.3%)	10.97 (3.42–132.32)	<0.0001
			Yes	39 (52%)	9 (12%)		
LYSHOLM Scale	<12	<0.0001 (0.0163)	No	1 (1.3%)	3 (4%)	3.94 (1.24–147.16)	0.0002
			Yes	70 (93.3%)	1 (1.3%)		
	>24		No	7 (9.3%)	20 (26.7%)	2.25 (1.07–8.21)	0.0085
			Yes	28 (37.3%)	20 (26.7%)		
KOOS Scale	<12	<0.0001 (0.0159)	No	0	4 (5.3%)	—-	<0.0001
			Yes	68 (90.7%)	3 (4%)		
	>24		No	3 (4%)	24 (32%)	3.19 (1.04–18.75)	0.0294
			Yes	17 (22.7%)	31 (41.3%)		

## Data Availability

The data underlying this article are available in the article and in its online Supplementary Materials.

## Supplementary Materials

The following supporting information can be downloaded at: https://www.mdpi.com/article/10.3390/jcm13144220/s1.
